# Monographs

**Published:** 1856-06

**Authors:** 


### ON THE ORGANIC DISEASES AND FUNCTIONAL DISORDERS OF THE STOMACH.

BY GEO. BUDD, M. D., F. R. S. &C., Blanchard & Lea, Philadelphia, 1856.

This is a late edition of a well known and standard work, with such additions and corrections as the author's experience suggested.

It is scarcely necessary at this late day, to say a word in commendation of a work so favorably received already, by the profession generally.

The plan of issuing monographs by competent men, is one of the great steps of improvement in medical science. Undistracted by the numerous subjects which at all times tax the observation of the general practitioner, the monographist concentrates his thoughts, brings to bear his knowledge of the opinions of others, and his own experience upon a given subject, and is thus enabled to amplify and illustrate more thoroughly than under other circumstances could well be done.

In this volume we have the most common and important class of diseases treated of in a most satisfactory manner.

The whole subject is brought quite up to the times, treated in a clear, full, scientific and highly practical way.

We must say we should suspect any physician of neglect or of indifference to the best interests of the sick, who neglects to consult Budd on Diseases of the Stomach.

It is only necessary to say that the publishers have gotten it up in their *usual* style, to commend all a publisher has to do with it.

